# Waking rest during retention facilitates memory consolidation, but so does social media use

**DOI:** 10.1038/s41598-025-88363-z

**Published:** 2025-02-18

**Authors:** Julian Quevedo Pütter, Edgar Erdfelder

**Affiliations:** University of Mannheim, School of Social Sciences, Mannheim, Germany

**Keywords:** Psychology, Human behaviour

## Abstract

A short period of post-encoding waking rest has been shown to benefit subsequent memory performance. For example, past research suggests that waking rest after learning Icelandic-German word pairs boosts subsequent recall relative to an equally long period of social media use. Such findings are typically interpreted as evidence in favor of *diversion retroactive interference*. According to this account, non-specific cognitive processing inhibits consolidation and thus impairs *storage* of information encoded previously. However, the effect might alternatively be explained by *similarity retroactive interference* according to which *retrieval* is hampered by information processed during retention. Here, we report two experiments that shed light on the mechanisms underlying the waking rest effect. In both experiments, participants either wakefully rested, used social media, or engaged in additional Norwegian-German vocabulary learning after the original learning phase. We performed multinomial processing tree (MPT) analyses to disentangle latent storage and retrieval contributions to cued recall and recognition performance. We did not find any memory differences between the waking rest and social media conditions in either experiment. Moreover, storage, but not retrieval, was reliably impaired in the vocabulary condition. Thereby, the present research provides direct behavioral evidence for a dominant role of consolidation in the waking rest effect.

## Introduction

Waking rest has been defined as a period of quiet, reflective thought void of distracting stimuli^1^. Superficially, such resting periods might appear rather unproductive or even a waste of time. However, quite to the contrary, waking rest has not only been linked to mental health and sleep benefits^1^, but is also argued to facilitate memory consolidation^2^. Indeed, a growing body of evidence suggests that wakefully resting after new learning can enhance subsequent memory performance compared to engaging in a cognitively demanding distractor task^2–4^.

Past research has used two alternative approaches to demonstrate the memory-enhancing effect of post-encoding waking rest. Some studies have compared conditions of equivalent levels of cognitive distraction (i.e., equivalent levels of retroactive interference) with different temporal positions of the distractor task during the retention interval^5,6^. Thus, participants in these studies engaged in the distractor task either immediately after learning or only after an interpolated period of waking rest. In contrast, the majority of studies has compared different levels of cognitive distraction such that the relative level of retroactive interference was either minimal (i.e., in the waking rest condition) or substantially increased (i.e., in a distractor condition) across the entire retention interval^3,4,7^.

In a recent study, Martini et al. ^7^ used the latter approach to demonstrate that social media use after learning new vocabulary can be detrimental to subsequent memory performance relative to a waking rest condition. More specifically, participants in this study learned and immediately recalled Icelandic-German word pairs, before being randomly assigned to either a waking rest or a social media condition. In the waking rest condition, participants rested for 8 minutes, whereas in the social media condition, participants used Facebook or Instagram for the same amount of time. In two delayed recall tests immediately after the 8-min retention interval and again after 24 hours, participants in the waking rest condition showed significantly less forgetting relative to the immediate recall than participants in the social media condition.

Given its simplicity, waking rest might be a promising behavioral intervention for improving memory in many applied settings^8–10^. However, such an optimistic perspective is challenged by a considerable number of studies that have failed to find significant effects of waking rest^11^. Thus, our first aim in the present research was to conduct a close replication of the social media study reported by Martini et al. ^7^. In our opinion, this study represents a particularly important replication target^12^ because of the combination of high practical relevance and rather low experimental control (i.e., largely unrestricted social media use) compared to other tasks that have been used before (e.g., spot-the-difference task^13^, further word pair learning^6^).

**Fig. 1:**
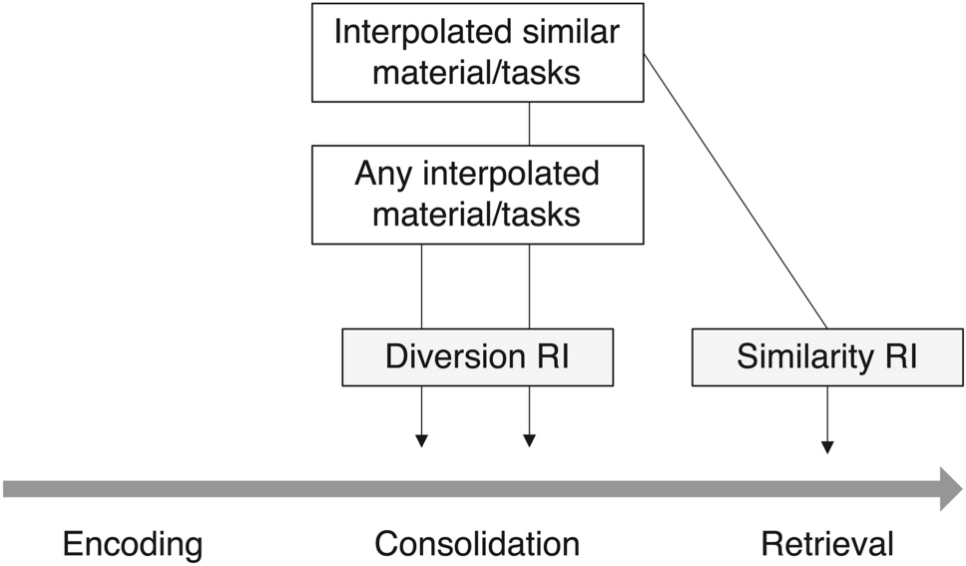
Illustration of the Dewar et al. theoretical model of retroactive interference^3^. Two types of retroactive interference are differentiated: diversion and similarity retroactive interference. First, any interpolated material or task will induce diversion retroactive interference and thereby interfere with memory consolidation. Second, only interpolated similar material and tasks will additionally induce similarity retroactive interference and thereby impair memory retrieval.

On a theoretical level, waking rest has been ascribed a central role in memory consolidation during wakefulness. According to the opportunistic theory of memory consolidation^14^, waking rest facilitates the consolidation of recently acquired memories by protecting limited hippocampal resources from retroactive interference. Conversely, any kind of distractor task that induces some minimal degree of cognitive processing or encoding demands should reduce the resources available for memory consolidation. Critically, this reasoning ignores possible contributions from similarity-based retroactive interference that might arise from similarities between the original learning material and the distractor task^15^. Indeed, Dewar et al.^3^ proposed an elegant theoretical model that differentiates two different types of retroactive interference: diversion retroactive interference and similarity retroactive interference (see Figure 1). On the one hand, diversion retroactive interference is assumed to be induced by any interpolated task or material regardless of its similarity to the original learning material. As long as the interpolated task or material relies at least to some extent on hippocampal resources, consolidation processes that rely on the same resources will be inhibited. On the other hand, similarity retroactive interference is only induced by similar tasks or materials and impairs retrieval of the target information due to reduced discriminability from the interfering information.

It follows that positive effects of post-encoding waking rest might represent an unknown combination of both diversion and similarity retroactive interference. In other words, differences between waking rest and distractor conditions that have been interpreted as evidence in favor of opportunistic consolidation might in many cases just as well be explained solely by retrieval differences or some unknown combination of both consolidation and retrieval processes. For example, in the study by Martini et al.^7^, participants in the social media condition might have engaged in posts that were semantically related to the previously studied word pairs. Thus, strictly speaking, there is currently no direct behavioral evidence for a role of consolidation in the waking rest effect.

Our second aim in the present research was to close this critical gap in the literature by using multinomial processing tree (MPT) modeling to precisely disentangle consolidation and retrieval contributions to memory performance^16,17^. Over the past decades, MPT models have been successfully applied in many different areas of psychology^17^. Storage-retrieval models represent a subset of MPT models that allow to decompose storage and retrieval contributions to memory performances.

**Fig. 2:**
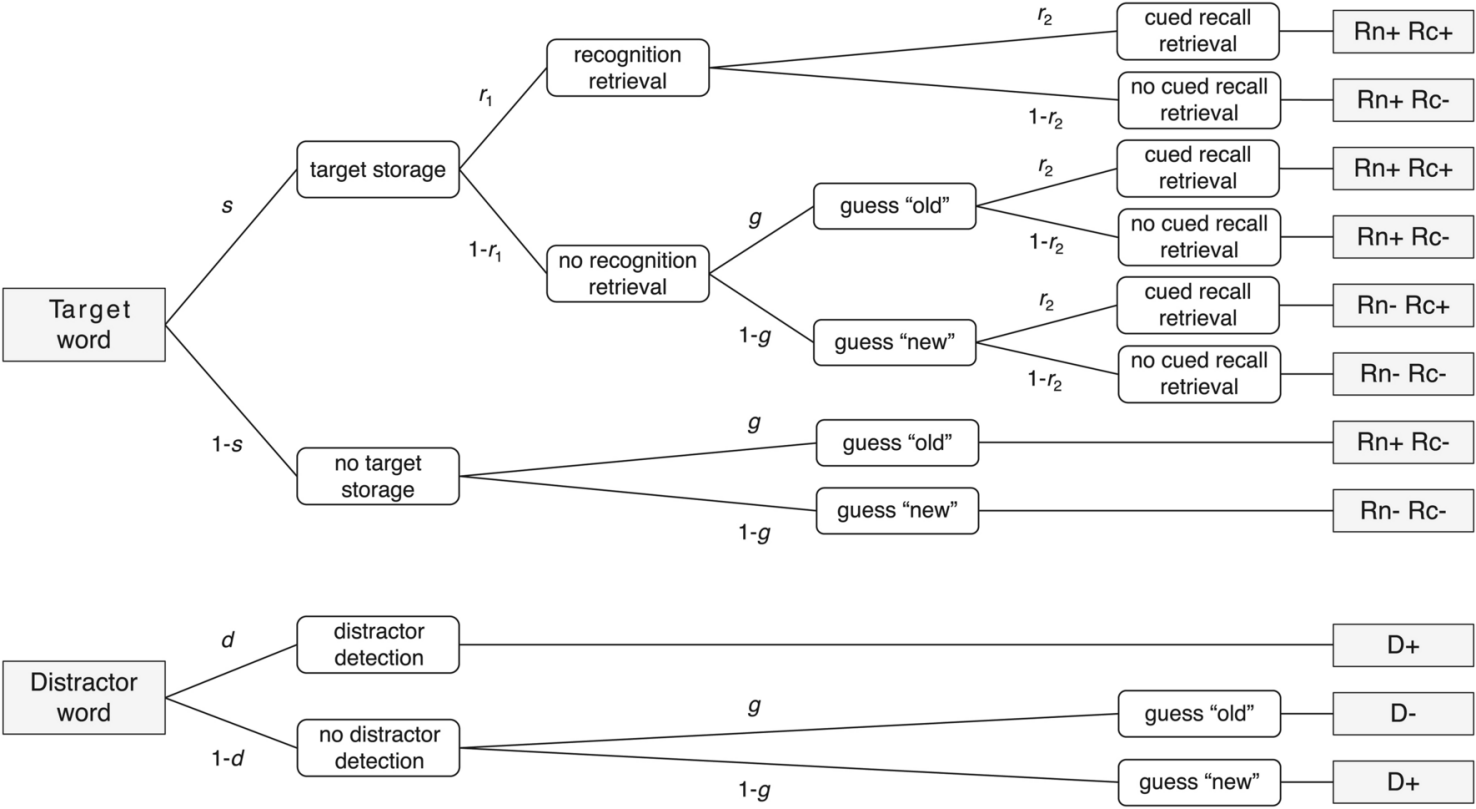
Illustration of the recognition-then-cued-recall storage-retrieval multinomial processing tree (MPT) model by Riefer and Batchelder^18,19^. Each branch of the processing tree represents one possible sequence of cognitive processes, reflected in the following parameters: *s* = probability of successful target storage, $r_1$ = probability of successful target retrieval during recognition, *g* = probability of guessing ’old’ during recognition, $r_2$ = probability of successful target retrieval during recall, *d* = probability of successful distractor detection during recognition. For a given target word, responses in the recognition and cued recall tests can be categorized as follows: Rn+ Rc+ = successful recognition and recall, Rn+ Rc- = successful recognition and unsuccessful recall, Rn- Rc+ = unsuccessful recognition and successful recall, Rn- Rc- = unsuccessful recognition and recall. For a given distractor word during recognition, D+ = correct rejection, D- = false alarm. Parameter *d* can be set equal to $s * r_1$^19^.

A storage-retrieval MPT model that is ideally suited for the paradigm used by Martini et al.^7^ was developed by Riefer and Batchelder^18^. It is tailored to a recognition-then-cued-recall paradigm for word pairs, that is, it allows for Icelandic-German vocabulary as to-be-learned material and only requires the inclusion of an additional old-new recognition test for the German target words (presented without their Icelandic cue words). Thereby, a given target word might fall into one of four possible response categories: successful recognition and cued recall (Rn+ Rc+), successful recognition and unsuccessful cued recall (Rn+ Rc-), unsuccessful recognition and successful cued recall (Rn- Rc+), or unsuccessful recognition and cued recall (Rn- Rc-). The probabilities of these response categories (which can be estimated from their observed frequencies) are reparameterized by means of a set of latent model parameters. Each parameter represents the probability of some cognitive processing step: First, a given target word will be successfully stored with probability $s$, whereas storage will fail with probability $1-s$. Next, a successfully stored target word will be retrieved versus not be retrieved during recognition with probabilities $r_1$ versus $1-r_1$, respectively. When retrieval during recognition fails, or a target word was not successfully stored in the first place, a participant will correctly guess ‘old’ versus incorrectly guess ‘new’ with probabilities $g$ versus $1-g$, respectively. Finally, a successfully stored target word will be retrieved versus not be retrieved during cued recall with probabilities $r_2$ versus $1-r_2$, respectively. The full tree structure of this model is illustrated in Figure 2.

Importantly, it directly follows from the structure of the Riefer and Batchelder model that parameters are reflective of memory processing steps relating to the target words only^19^. Specifically, parameter $s$ reflects target (but not associative) storage because the model does not account for recognition retrieval of cue-target associations. In line with this, the recognition test includes target words only and ignores cue words, so parameter $r_1$ can be assumed to reflect recognition retrieval of target words (but not cue words or cue-target associations)^19^. Thus, the main focus of this research was on item rather than associative memory^20,21^, although cued recall performances may be assumed to be indicative of associative memory to some extent as well.

Parameters $s$ and $r_2$ of this MPT model can be related to diversion and similarity retroactive interference in a straightforward manner. First, parameter $s$ represents the probability of a target word being stored in memory across the retention interval. Thereby, it represents a combination of successful encoding and consolidation contributions. Because the experimental manipulation in waking rest studies takes place only after the learning phase, it cannot affect encoding. Thus, differences in parameter $s$ between conditions mirror differences in storage due to diversion retroactive interference. Second, parameter $r_2$ represents the probability of a stored target word being retrievable during cued recall. Accordingly, it should reflect effects of similarity retroactive interference. Note that the recognition retrieval parameter $r_1$ is not of substantive interest here. In fact, the probability of retrieving a stored target in an old-new recognition test should always be close to 1.

The model version originally proposed by Riefer and Batchelder^18^ can be adapted for our current purpose. First, memory performance for distractor items in the recognition test can be included in the model^19^. A distractor word always falls in one of two possible response categories: correct rejection (D+) or false alarm (D-). Parameter $d$ represents the probability of successfully detecting a distractor word during recognition. By assuming this probability to be equal to the probability of successfully retrieving a stored target word during recognition (i.e., $d = s * r_1$, as proposed by Nadarevic^19^), inclusion of distractor words into the model yields a saturated model version with four non-redundant category frequencies ($4-1 = 3$ for the target words plus $2-1 = 1$ for the distractor words) and four to-be-estimated parameters ($s$, $r_1$, $g$, $r_2$). This model version is identifiable but not testable since its goodness-of-fit test has *df* $= 4 - 4 = 0$. By introducing further equality constraints between experimental conditions, testable model versions with *df* $> 0$ can be defined that allow for model fit evaluations within a maximum likelihood (ML) framework^22^.

Second, the original recognition-then-cued-recall procedure can be replaced by a reversed cued-recall-then-recognition test sequence. The corresponding cued-recall-then-recognition storage-retrieval MPT model is mathematically equivalent to the original model but remedies the potential problem of the original procedure that the delayed cued recall performance might be positively biased by target presentation in the preceding old-new recognition test (perhaps serving as an additional learning occasion).

In the present research, we conducted two lab experiments to evaluate the replicability of the waking rest versus social media effect found by Martini and collaborators^7^, and to disentangle the contributions of diversion and similarity retroactive interference mediated by storage and retrieval effects, respectively. Both studies mainly differed in the order of memory tests, that is, Experiment 1 involved the original recognition-then-cued-recall testing procedure, whereas Experiment 2 involved a reversed cued-recall-then-recognition procedure. Thereby, in Experiment 1, we exactly followed the procedure for which the storage-retrieval model by Riefer and Batchelder^18^ had originally been developed, whereas in Experiment 2, the procedure was adjusted to more closely replicate the Martini et al.^7^ paradigm.

In both studies, we extended the original study design by including a vocabulary condition in which participants engaged in an intentional learning task. More specifically, this third group of participants was presented with an additional list of Norwegian-German word pairs^6^. Thereby, our design included not only a low similarity (i.e., social media) but also a high similarity (i.e., vocabulary) distractor condition. Thus, based on the Dewar et al. theoretical model^3^, we expected similarity retroactive interference to be lowest in the waking rest and highest in the vocabulary condition. Indeed, potential similarities between original and interpolated learning should have been most pronounced in the vocabulary condition due to semantic overlap between German target words from both lists and perceived phonetic overlap between Norwegian and Icelandic cue words for participants unfamiliar with these two Scandinavian languages. Whereas uncontrolled social media content may also have included some semantic overlap with the original learning material, such similarities could be expected to be effectively minimized in the waking rest condition.

In addition, we expected diversion retroactive interference to be lowest in the waking rest condition but not to differ necessarily between the social media and the vocabulary condition. Indeed, any nonspecific cognitive processing and encoding demands are generally expected to be minimal under conditions of waking rest^3^. Conversely, it was unclear whether or not the strong sensory input to be expected during social media use and the high encoding demands during intentional vocabulary learning would induce different degrees of diversion retroactive interference. Thus, we refrained from specifying directional hypotheses with respect to differences in diversion retroactive interference between the social media and the vocabulary condition.

Our hypotheses, study protocols, and analysis plans for both studies were preregistered on the Open Science Framework (OSF)^23,24^.

## Experiment 1

### Methods

#### Participants

We conducted an a priori power analysis using G*Power^25^ with the aim to obtain a statistical power of $1-\beta = 80\%$ to detect a medium effect size of Cohen’s $f = 0.25$ with $\alpha = 5\%$ in a one-factorial between-participants ANOVA model with three experimental groups. This setup yielded a required sample size of *N* = 159 participants (i.e., *n* = 53 per group).

Participants were recruited at the University of Mannheim. Interested individuals were eligible for participation only if (a) their first language was German or they were fluent in German, (b) they were between 18 and 32 years old (this corresponds to the age range in the sample from the original Martini et al.^7^ study), (c) they did not know any Icelandic or Norwegian, and (d) they had a smartphone with the Instagram app installed. Participants were randomly assigned to one of the experimental conditions using block randomization to ensure equal group sizes. All participants received course credit for an estimated net study duration of 45 minutes. In line with the original report by Martini et al., no further exclusion criteria were preregistered.

We collected data from 159 participants. Mean age in the full sample was 22.74 years (*SD* = 2.50, *range* = 18-31). 109 participants (68.55%) indicated to be female, 49 participants (30.82%) indicated to be male, and one participant identified their gender as non-binary. 154 participants (96.86%) indicated German to be their first language, and all participants confirmed to be fluent in German. 143 participants (89.94%) were studying at the time of participation, with 99 participants (62.26%) being enrolled in a psychology program.

The study was conducted in accordance with the Declaration of Helsinki (2013). As the study did not involve deception or other ethically relevant elements, formal approval from the ethics committee was not necessary according to the regulations of the ethics committee of the University of Mannheim. Informed consent was obtained from all participants.

#### Design

We used a between-participants manipulation of post-encoding activity with three levels: waking rest (minimal diversion and similarity retroactive interference), social media (high diversion and low similarity retroactive interference), and vocabulary (high diversion and similarity retroactive interference).

#### Material

To generate Icelandic-German word pairs for the original learning phase, Norwegian-German word pairs for the interpolated learning phase in the vocabulary condition, and German distractor words for the first and second delayed recognition tests, 84 German 5-letter nouns were selected from a word pool provided by Dimigen et al.^26^. For the 24 Icelandic-German word pairs, German words were chosen such that their Icelandic translations did not resemble their German or English translations. For the 20 Norwegian-German word pairs, the same rule was applied. We opted against using the original Martini et al.^7^ Icelandic-German word pairs due to the necessity to generate an additional pool of 20 distractor words per recognition test that shared the characteristics of the target words.

#### Procedure

The procedure of our study included two experimental sessions separated by about 24 hours. Both sessions took place in the same laboratory room equipped with movable walls between work stations. Participants were told that our research aim was to investigate leisure activities of university students.

**Session 1** First, participants provided informed consent and were checked for inclusion criteria. To comply as closely as possible with the original procedure, participants were then asked to report their current arousal and valence levels on a 7-point Likert scale. Next, during the learning phase, participants were presented with 24 Icelandic-German word pairs. They were told to memorize the material for a vocabulary test that would follow immediately. Each word pair was presented for 12 seconds and with a 3-sec inter-stimulus interval (ISI) in white font on a black background. Whereas the first 20 word pairs were presented in randomized order, the last 4 word pairs served as buffer items, that is, they were always presented in the same order and were not included in any memory tests. For the immediate cued recall, all 20 Icelandic cue words were presented simultaneously on the computer screen. Participants were given 3 minutes to type in as many of the previously learned German target words as possible. In contrast to the original procedure by Martini et al.^7^, we applied a learning criterion of 35% (i.e., at least 7 correct responses) to ensure sufficiently high frequencies in all response categories of the storage-retrieval MPT model. Specifically, the structure of the target tree of the model implies that rather low encoding rates would lead to relatively high frequencies in the categories resulting from the two lowermost branches (i.e., the two branches including no target storage), but relatively low frequencies in the categories resulting from the remaining branches (i.e., the six branches including successful target storage). Crucially, retrieval parameters $r_1$ and $r_2$ are only included in the six uppermost branches. Thus, rather high encoding rates were necessary to allow for sufficiently precise retrieval parameter estimates. Similar approaches have been used in other storage-retrieval MPT applications before^27,28^. If a participant failed to reach the 35% learning criterion during the first study-test cycle, they repeated this part of the procedure up to two additional times. Participants who did not reach the learning criterion in any repetition were excluded from further participation.

Next, an 8-min retention interval followed, during which participants engaged in their respective post-encoding activity. Participants in the waking rest and social media conditions were provided with headphones to minimize acoustic distractions. In the waking rest condition, they were instructed to relax as much as possible, but not to fall asleep. They were asked to lay their heads on their arms and to close their eyes. In the social media condition, participants were asked to engage in as many Instagram posts as possible on their own smartphones, but not to submit any own posts and not to follow any external links. They were also asked to use Instagram without tone to not distract other participants. During the entire experimental procedure, the shutters were closed and the lights turned off for all conditions. After 8 minutes had passed, participants in the waking rest and social media conditions received an acoustic signal over the headphones. In the vocabulary condition, participants learned and immediately recalled 20 Norwegian-German word pairs for 8 minutes. None of the German target words was previously included in the original learning phase. The procedure was the same as in the original learning phase, but there were no buffer items and no learning criterion was used.

After their respective post-encoding activity, all participants were again asked for their current arousal and valence levels. Afterwards, participants engaged in a first surprise delayed recognition-then-cued-recall test procedure. In the recognition test, participants were presented with a randomized sequence of 40 German words (i.e., 20 ‘old’ target and 20 ‘new’ distractor words). They were asked to indicate for each word whether it was ‘old’ (i.e., previously presented as part of an Icelandic-German word pair) by pressing the ‘S’ key on their keyboard or ‘new’ (i.e., not previously presented) by pressing the ‘L’ key. No time limit was imposed during recognition. The first delayed cued recall test was identical to the immediate cued recall test.

To conclude Session 1, participants were instructed to answer questions concerning thoughts about and conscious rehearsal of the Icelandic-German word pairs during the 8-min retention interval. In the social media condition, participants were additionally asked to estimate the total number of Instagram posts and the number of Instagram posts in Icelandic language they had engaged in.

**Session 2** The procedure of Session 2 encompassed a second surprise delayed recognition-then-cued-recall test procedure that was identical to the first delayed test procedure from Session 1, except for a new set of 20 distractor words in the recognition test. The purpose of this second delayed testing session was to evaluate whether any short-term effects of our manipulation on recognition and cued recall performances would persist or even increase over a longer time scale of 24 hours as had been found by Martini et al. ^7^. Finally, participants were asked for information concerning the 24-hr interval between sessions, including sleep times, alcohol consumption, and the same questions concerning thoughts about and active rehearsal of the Icelandic vocabulary as in Session 1. After providing demographic information, participants were thanked and debriefed.

#### Data analysis

All analyses were conducted in R^29^. A significance level of $\alpha$ = 5% was used for all analyses.

We computed cued recall retention and recognition performance scores as dependent variables. For the cued recall retention scores, the number of correct responses in the respective delayed cued recall test was divided by the number of correct responses in the immediate cued recall test in which the learning criterion was reached for each participant. For the recognition performance score, false-alarm rates were subtracted from hit rates to obtain a response-bias-corrected recognition measure per participant^19^.

Cued recall and recognition differences between conditions were analyzed by means of one-factorial between-participants ANOVA. These were followed up by multiple planned contrasts to infer the significance of the pairwise differences between the respective conditions of interest^30^.

We obtained MPT parameter estimates from two different estimation approaches to ensure the robustness of our model-based conclusions. First, using response category frequencies aggregated within experimental conditions, we applied an ML estimation approach as implemented in the R package MPTinR^31^. Second, for the individual response category frequencies, we applied the Bayesian hierarchical latent-trait estimation approach^32^ as implemented in the R package TreeBUGS^33^. Whereas the first approach rests on the assumption of identically and independently distributed (i.i.d.) observations, the latter takes into account the potential heterogeneity of participants and includes parameter correlations^33^.

For the aggregated data, the model was estimated simultaneously for all three experimental conditions. By applying equality constraints between conditions, a model version with *df* > 0 was obtained that allowed for testing the model via the $G^2$ goodness-of-fit statistic. For the individual data, the model was estimated separately for each experimental condition. Convergence of the MCMC sampler was confirmed according to the potential scale reduction factor $\hat{R} < 1.05$^34^. Model fit was evaluated with respect to the posterior-predictive *p*-values obtained from comparing the fit statistics $T_1$ and $T_2$ for the observed and posterior-predicted data^32^. $p_{T_1}$ and $p_{T_2}$ values $> 0.05$ are considered to reflect satisfactory model fit^33^.

For the aggregated data, equality constraints were imposed on the respective parameters of interest, and the significance of parameter differences between experimental conditions was inferred from the reduction in model fit, that is, $\Delta G^2$ in the case of two-tailed research questions, and $z = \sqrt{\Delta G^2(1)}$ the case of one-tailed hypotheses. For the individual data, posterior distributions of parameter differences were used to infer the reliability of parameter differences between conditions^17^. More specifically, for our preregistered one-tailed hypotheses, so-called Bayesian *p*-values were calculated, that is, the relative amount of the posterior distribution below zero. For two-tailed research questions, 95% Bayesian credibility intervals (BCI) were used as a reliability criterion. Given that aggregated and individual MPT results converged in most cases (as has previously been observed for a range of different MPT models^35^ and is also expected for models of the type relevant here^36^), Bayesian *p*-values and 95% BCI are only reported when inferences differed from those obtained for the aggregated data. The remaining values are provided in the Supplementary Material.

### Results

#### Manipulation check

The mean number of Instagram posts that participants in the social media condition reported to have engaged in was *M* = 23.28 (*SD* = 13.15, *range* = 6-70). Only one participant reported to have engaged in any Icelandic posts. The mean number of correct responses in the interpolated cued recall in the vocabulary condition was *M* = 12.43 (*SD* = 4.70).

**Table 1:** Mean (*SD*) cued recall and recognition performances in Session 1 of Experiment 1. A total of 20 word pairs was presented to participants during the original learning phase. The learning phase and the immediate cued recall were presented between one and three times to participants. Recall retention = correct responses in delayed cued recall / correct responses in immediate cued recall. Recognition performance = hit rate − false-alarm rate.

Measure	Waking rest	Social media	Vocabulary
Immediate recall: Repetitions	1.39 (0.53)	1.48 (0.50)	1.38 (0.60)
Immediate recall: Correct responses (absolute)	11.59 (2.66)	11.94 (3.56)	10.96 (2.69)
Immediate recall: Correct responses (proportion)	0.58 (0.13)	0.60 (0.18)	0.55 (0.13)
First delayed recall: Correct responses (absolute)	13.39 (3.11)	13.46 (3.98)	11.81 (3.37)
First delayed recall: Correct responses (proportion)	0.67 (0.16)	0.67 (0.20)	0.59 (0.17)
First delayed recall: Retention	1.16 (0.15)	1.14 (0.18)	1.08 (0.17)
First delayed recognition: Hit rate	0.95 (0.08)	0.94 (0.07)	0.88 (0.11)
First delayed recognition: False-alarm rate	0.05 (0.06)	0.04 (0.05)	0.04 (0.06)
First delayed recognition: Performance	0.90 (0.10)	0.91 (0.09)	0.83 (0.13)

#### Cued recall and recognition

An inspection of cued recall and recognition measures in the full sample revealed some rather severe outliers in both Sessions 1 and 2. To avoid biased results while not overly compromising the statistical power of our hypothesis tests, we decided to deviate from our preregistered analysis plan by applying a conservative outlier criterion and excluding extreme values from both sessions separately. We excluded participants whose cued recall retention or recognition performance score was more than three times the median absolute distance (MAD) away from the respective grand median^37^. This approach resulted in sample sizes of $N_1$ = 154 for Session 1 (*n* = 51 in the waking rest condition, *n* = 50 in the social media condition, *n* = 53 in the vocabulary condition) and $N_2$ = 141 for Session 2 (*n* = 49 in the waking rest and social media conditions, *n* = 43 in the vocabulary condition). The resulting descriptive statistics for the cued recall and recognition measures in Session 1 are provided in Table 1.

The number of repetitions of the immediate cued recall necessary to reach the learning criterion did not differ significantly between conditions, *F*(2, 151) = 0.52, *p* = 0.593, $\eta ^2$ = 0.01. The same was true for the number of correct responses in the immediate cued recall in which the learning criterion was reached, *F*(2, 151) = 1.42, *p* = 0.245, $\eta ^2$ = 0.02.

We hypothesized that cued recall retention in the first delayed recall test would be higher in the waking rest condition than in the social media and vocabulary conditions, respectively. Overall, there was a significant effect of our manipulation on the number of correct responses in the first delayed cued recall, *F*(2, 151) = 3.71, *p* = 0.027, $\eta ^2$ = 0.05. More importantly, in line with our hypotheses, cued recall retention did differ significantly between conditions as well, *F*(2, 151) = 3.48, *p* = 0.033, $\eta ^2$ = 0.04. We performed planned contrasts to further evaluate our hypotheses. As expected, cued recall retention was significantly higher in the waking rest than in the vocabulary condition, *t*(151) = 2.55, *p* = 0.006, Cohen’s *d* = 0.50. However, this was not the case when the waking rest condition was compared against the social media condition, *t*(151) = 0.71, *p* = 0.238, Cohen’s *d* = 0.14. There was also no significant difference (two-tailed) between the social media and the vocabulary condition, *t*(151) = 1.82, *p* = 0.071, Cohen’s *d* = 0.36.

We also hypothesized that performance in the first delayed recognition test would be higher in the waking rest condition than in the social media and the vocabulary conditions, respectively. There again was an overall effect of our manipulation, *F*(2, 151) = 7.41, *p* = 0.001, $\eta ^2$ = 0.09. Mirroring our findings for cued recall retention, planned contrasts revealed that recognition performances were significantly lower in the vocabulary than in the waking rest condition, *t*(151) = 3.15, *p* = 0.001, Cohen’s *d* = 0.62, whereas recognition performances in the social media condition did not differ significantly from those in the waking rest condition, *t*(151) = -0.33, *p* = 0.627, Cohen’s *d* = -0.06. In line with this pattern, there was a significant difference (two-tailed) between the social media and the vocabulary condition, *t*(151) = 3.47, *p* < 0.001, Cohen’s *d* = 0.68.

Descriptive statistics for Session 2 are provided in Supplementary Table S1. Overall, no significant effects emerged between the waking rest and the social media condition, and the differences between the waking rest and the vocabulary condition were substantially reduced. Indeed, only the effect on cued recall retention remained significant, *t*(138) = 1.88, *p* = 0.031, Cohen’s *d* = 0.39.

We confirmed the robustness of our main conclusions concerning cued recall and recognition measures in a sensitivity analysis (see Supplementary Tables S3 and S5).

**Table 2:** Storage-retrieval multinomial processing tree (MPT) parameter estimates [95% CI] in Session 1 of Experiment 1. Parameter *s* = probability of successful target storage, $r_1$ = probability of successful target retrieval during recognition, *g* = probability of guessing ’old’ during recognition, $r_2$ = probability of successful target retrieval during recall. For the aggregated data, the model was fitted using ML estimation in the R package MPTinR^31^ (95% confidence intervals in brackets), and parameter $r_1$ was set equal between the waking rest and the social media condition to allow for a model fit evaluation. For the individual data, the model was fitted using Bayesian hierarchical estimation in the R package TreeBUGS^33^ (95% Bayesian credibility intervals in brackets).

Parameter	Waking rest	Social media	Vocabulary
Aggregated data			
Storage (*s*)	0.91 [0.90, 0.93]	0.92 [0.91, 0.94]	0.87 [0.84, 0.89]
Recognition retrieval ($r_1$)	0.98 [0.97, 0.99]	0.98 [0.97, 0.99]	0.96 [0.94, 0.98]
Guessing “old” (*g*)	0.46 [0.36, 0.55]	0.38 [0.28, 0.48]	0.27 [0.20, 0.33]
Cued recall retrieval ($r_2$)	0.73 [0.70, 0.76]	0.73 [0.70, 0.76]	0.68 [0.65, 0.71]
Individual data			
Storage (*s*)	0.94 [0.91, 0.97]	0.94 [0.91, 0.97]	0.89 [0.85, 0.93]
Recognition retrieval ($r_1$)	0.99 [0.97, 1.00]	0.98 [0.97, 1.00]	0.99 [0.97, 1.00]
Guessing “old” (*g*)	0.57 [0.37, 0.80]	0.36 [0.20, 0.52]	0.21 [0.10, 0.32]
Cued recall retrieval ($r_2$)	0.74 [0.69, 0.78]	0.75 [0.68, 0.81]	0.69 [0.63, 0.74]

#### Storage-retrieval MPT model probabilities

Our preregistered MPT model specification included an equality constraint on guessing parameter *g* across conditions for the aggregated data. However, this model version did not fit the data, neither for Session 1, *G*^2^(2) = 11.34, *p* = 0.003, nor for Session 2, $G^2$(2) = 12.11, *p* = 0.002. Instead, an inspection of parameter estimates resulting from a saturated model version indicated that recognition retrieval parameters $r_1$ might be similar enough between conditions to allow for an equality constraint, especially between the waking rest and social media conditions. Indeed, such a model version fit the data well both for Session 1, $G^2$(1) = 0.42, *p* = 0.515, and for Session 2, $G^2$(1) = 0.15, *p* = 0.703. Thus, we used this model version for further MPT analyses. For the individual data, good convergence was observed for all parameters in all three conditions, all $\hat{R} < 1.05$, and the model fit the data well in both sessions.

MPT parameter estimates for Session 1 are provided in Table 2. Estimates largely aligned between both estimation approaches for the aggregated and individual data. Based on the assumption of diversion retroactive interference, we expected MPT storage probabilities $s$ in Session 1 to be higher in the waking rest than in the social media and vocabulary conditions, respectively. We also hypothesized that storage probabilities would not differ significantly between the social media and the vocabulary conditions because considerable cognitive processing demands should have been induced in both conditions (see Introduction). Contrary to our hypothesis, there even was a slight descriptive tendency of higher storage probabilities in the social media than in the waking rest condition, $z$ = 0.73, *p* = 0.767. In contrast, storage probabilities were significantly higher in the waking rest compared to the vocabulary condition, $z$ = 2.99, *p* = 0.001. Against our expectations, there was also a significant difference in storage probabilities between the social media and the vocabulary condition, $\Delta G^2(1)$ = 13.40, *p* < 0.001.

Based on the assumption of similarity retroactive interference, we hypothesized that cued recall retrieval probabilities $r_2$ in Session 1 would be higher in the waking rest than in the social media and the vocabulary conditions, respectively, and also in the social media compared to the vocabulary condition (see Introduction). Against our expectations, cued recall retrieval probabilities in Session 1 did not differ significantly between the waking rest and the social media condition, $z$ = 0.20, *p* = 0.421. For the remaining pairwise comparisons, the results were rather mixed: For the individual data, cued recall retrieval probabilities were not reliably higher in the waking rest than in the vocabulary condition, Bayesian *p* = 0.096, or in the social media than in the vocabulary condition, Bayesian *p* = 0.082. In contrast, for the aggregated data, these comparisons yielded significantly higher cued recall retrieval probabilities in the waking rest than in the vocabulary condition, $z$ = 2.21, *p* = 0.014, and in the social media than in the vocabulary condition, $z$ = 2.03, *p* = 0.021.

With respect to the remaining parameters, recognition retrieval probabilities $r_1$ were estimated to be very close to 1 in all three conditions. For guessing probabilities $g$, we observed a descriptive reduction of the probability to guess ‘old’ from the waking rest to the vocabulary condition. Indeed, guessing probabilities did differ significantly between the waking rest and the vocabulary condition, $\Delta G^2(1)$ = 10.65, *p* = 0.001. However, this was not the case for the comparisons between the waking rest and social media conditions, $\Delta G^2(1)$ = 1.16, *p* = 0.282, or the social media and vocabulary conditions, $\Delta G^2(1)$ = 3.80, *p* = 0.051.

MPT parameter estimates for Session 2 are provided in Supplementary Table S2. Whereas storage probabilities $s$ did not differ significantly between conditions anymore, the patterns for parameters $r_1$, $r_2$ and $g$ were very similar to those from Session 1.

We confirmed the robustness of our MPT results in a sensitivity analysis (see Supplementary Tables S4 and S6).

## Experiment 2

### Methods

Our methodological approach in Experiment 2 largely followed that of Experiment 1, except for a reversal of the recognition-then-cued-recall test procedure and some other deviations and extensions detailed below. Thereby, Experiment 2 allowed for an even closer replication of the original Martini et al.^7^ study because the additional recognition test took place only *after* the delayed cued recall test. Thus, our aim in Experiment 2 was to evaluate whether our failed replication of the waking rest versus social media effect in Experiment 1 was due to some unexpected influence of the interpolated recognition test on subsequent cued recall performances.

#### Participants

Our sample size rationale was the same as in Experiment 1, that is, we aimed at a statistical power of $1-\beta = 80\%$ to detect a medium effect size of Cohen’s $f = 0.25$ with $\alpha = 5\%$. However, after observing some rather severe outliers with respect to cued recall retention and recognition performance scores in Experiment 1, we oversampled by 10% to account for the necessity to exclude outliers. Thus, the required sample size was *N* = 177 participants (i.e., *n* = 59 per condition). We preregistered the same exclusion criterion we already used in Experiment 1, that is, participants whose cued recall retention or recognition performance score was more than three times the MAD away from the respective grand median were excluded^37^.

Participants were again recruited at the University of Mannheim. The same eligibility requirements applied as in Experiment 1, and the same 35% learning criterion was used in the immediate cued recall. Participants could choose between course credit and a financial compensation of 10€ for an estimated net study duration of 45 minutes within one single session.

We collected data from 177 participants. After excluding participants who failed to reach the 35% learning criterion in the immediate cued recall or were identified as outliers according to their recall retention or recognition performance score, the final sample size was *N* = 157 (*n* = 53 in the waking rest condition, *n* = 52 in the social media and vocabulary conditions). Mean age was 22.17 years (*SD* = 2.98, *range* = 18-31). 117 participants (74.52%) indicated to be female, 37 participants (23.57%) indicated to be male, one participant identified their gender as non-binary, and one participant as diverse. One participant refrained from providing their gender identity. 144 participants (91.72%) indicated German to be their first language. As in Experiment 1, all participants confirmed to be fluent in German. 149 participants (94.90%) were studying at the time of participation, and 79 participants (50.32%) were enrolled in a psychology program.

The study was again conducted in accordance with the Declaration of Helsinki (2013). As the study did not involve deception or other ethically relevant elements, formal approval from the ethics committee was not necessary according the regulations of the ethics committee of the University of Mannheim. Informed consent was obtained from all participants.

#### Procedure

In contrast to Experiment 1, we decided to focus on short-term effects of post-encoding waking rest within a single session. Thus, our procedure in Experiment 2 was the same as in Session 1 of Experiment 1. The delayed testing procedure was reversed, that is, instead of a recognition-then-cued-recall procedure as in Experiment 1, we used a cued-recall-then-recognition procedure. To further increase the sensory input during the 8-min retention interval in the social media condition, participants were asked to bring headphones with them so that they could use Instagram with tone.

We used the German translation of the High Sensitive Person Scale (HSPS-G)^38,39^ and the negative emotionality subscale of the German version of the Big Five Inventory 2 (BFI-2)^40^ as part of a post-experimental questionnaire to assess sensory processing sensitivity and neuroticism as potential moderators of the waking rest effect^41^. However, these covariates were not of interest in our present research and will be used in separate analyses not reported here.

### Results

#### Manipulation check

The mean number of Instagram posts that participants in the social media condition reported to have engaged in was *M* = 24.13 (*SD* = 21.39, *range* = 6-150). Two participants reported to have engaged in any Icelandic posts. The mean number of correct responses in the interpolated cued recall in the vocabulary condition was *M* = 13.58 (*SD* = 4.14).

**Table 3:** Mean (*SD*) cued recall and recognition performances in Experiment 2. A total of 20 word pairs was presented to participants during the original learning phase. The learning phase and the immediate cued recall were presented between one and three times to participants. Recall retention = correct responses in delayed cued recall / correct responses in immediate cued recall. Recognition performance = hit rate − false-alarm rate.

Measure	Waking rest	Social media	Vocabulary
Immediate recall: Repetitions	1.49 (0.58)	1.52 (0.58)	1.40 (0.63)
Immediate recall: Correct responses (absolute)	11.42 (2.95)	12.00 (2.90)	11.62 (2.92)
Immediate recall: Correct responses (proportion)	0.57 (0.15)	0.60 (0.15)	0.58 (0.15)
Delayed recall: Correct responses (absolute)	11.62 (3.03)	12.17 (2.85)	11.25 (3.39)
Delayed recall: Correct responses (proportion)	0.58 (0.15)	0.61 (0.14)	0.56 (0.17)
Delayed recall: Retention	1.02 (0.10)	1.02 (0.13)	0.96 (0.11)
Delayed recognition: Hit rate	0.95 (0.05)	0.94 (0.07)	0.91 (0.08)
Delayed recognition: False-alarm rate	0.03 (0.06)	0.02 (0.04)	0.03 (0.04)
Delayed recognition: Performance	0.92 (0.07)	0.92 (0.09)	0.88 (0.09)

#### Cued recall and recognition

Descriptive statistics for cued recall and recognition measures are provided in Table 3. The number of repetitions of the immediate cued recall did not differ significantly between conditions, *F*(2, 154) = 0.53, *p* = 0.591, $\eta ^2$ = 0.01. The same was true for the number of correct responses in the respective last immediate cued recall, *F*(2, 154) = 0.54, *p* = 0.584, $\eta ^2$ = 0.01.

We hypothesized that cued recall retention in the delayed recall test would be higher in the waking rest condition than in the social media and the vocabulary conditions, respectively. Somewhat surprisingly, the number of correct responses in the delayed cued recall did not differ significantly between conditions, *F*(2, 154) = 1.17, *p* = 0.313, $\eta ^2$ = 0.01. Nevertheless, in line with our preregistered hypotheses, an ANOVA did reveal a significant overall effect of our manipulation on cued recall retention, *F*(2, 154) = 4.98, *p* = 0.008, $\eta ^2$ = 0.06. Planned contrasts showed that cued recall retention was significantly higher in the waking rest condition compared to the vocabulary condition, *t*(154) = 2.70, *p* = 0.004, Cohen’s *d* = 0.53, but not compared to the social media condition, *t*(154) = -0.09, *p* = 0.534, Cohen’s *d* = -0.02. There was a significant difference (two-tailed) between the social media and the vocabulary condition, *t*(154) = 2.77, *p* = 0.006, Cohen’s *d* = 0.54.

We also hypothesized that performance in the delayed recognition test would be higher in the waking rest condition than in the social media and the vocabulary conditions, respectively. Again, an ANOVA revealed a significant effect of our manipulation, *F*(2, 154) = 3.61, *p* = 0.029, $\eta ^2$ = 0.04. In line with the pattern for cued recall retention, this overall effect could be attributed to significantly lower recognition performances in the vocabulary than in the waking rest condition, *t*(154) = 2.23, *p* = 0.014, Cohen’s *d* = 0.43, whereas recognition performances in the social media condition did not differ significantly from those in the waking rest condition, *t*(154) = -0.20, *p* = 0.579, Cohen’s *d* = -0.04. There was a significant difference between the social media and the vocabulary condition (two-tailed), *t*(154) = 2.42, *p* = 0.017, Cohen’s *d* = 0.47.

As in Experiment 1, we confirmed the robustness of our main conclusions concerning cued recall and recognition measures in a sensitivity analysis (see Supplementary Table S7).

#### Storage-retrieval MPT model probabilities

MPT parameter estimates are provided in Table 4. Again, estimates largely aligned between both estimation approaches. For the aggregated data, the same baseline model that we already used in Experiment 1 fit the data well, $G^2$(1) = 1.65, *p* = 0.199. For the individual data, good convergence was observed for all parameters in all three conditions, all $\hat{R} < 1.05$, and the model fit the data well.

We hypothesized that MPT storage probabilities $s$ would be higher in the waking rest than in the social media and vocabulary conditions, respectively (see Introduction). As in Experiment 1, there instead was a descriptive tendency of higher storage probabilities in the social media compared to the waking rest condition, $z$ = 0.17, *p* = 0.568. In contrast, storage probabilities in the vocabulary condition were significantly smaller than in the waking rest condition, $z$ = 2.25, *p* = 0.012. In line with these two findings, storage probabilities in the vocabulary condition were also significantly smaller than in the social media condition, $\Delta G^2(1)$ = 5.83, *p* = 0.016.

We expected MPT cued recall retrieval probabilities $r_2$ to be higher in the waking rest than in the social media and the vocabulary conditions, respectively, and also in the social media compared to the vocabulary condition (see Introduction). As we had observed for parameter $s$, we found a descriptive tendency of higher cued recall retrieval probabilities in the social media compared to the waking rest condition, $z$ = 1.25, *p* = 0.895. Surprisingly, cued recall retrieval probabilities in the vocabulary condition were not significantly smaller than in the waking rest condition either, $z$ = 0.05, *p* = 0.518. The same held true for the comparison of the social media and the vocabulary condition, $z$ = 1.19, *p* = 0.117.

As for the remaining parameters, recognition retrieval probabilities $r_1$ were estimated to be very close to 1. With respect to guessing probabilities $g$, the pattern was more complex: For the individual data, no reliable differences were observed between any of the three conditions, that is, all pairwise 95% BCI overlapped zero. In contrast, for the aggregated data, guessing probabilities were estimated to be significantly higher in the waking rest compared to the social media condition, $\Delta G^2(1)$ = 4.02, *p* = 0.045, and also compared to the vocabulary condition, $\Delta G^2(1)$ = 4.77, *p* = 0.029. Estimates did not differ significantly between the social media and the vocabulary condition, $\Delta G^2(1)$ = 0.00, *p* = 0.981.

As in Experiment 1, we confirmed the robustness of our MPT results in a sensitivity analysis (see Supplementary Table S8).

**Table 4:** Storage-retrieval multinomial processing tree (MPT) parameter estimates [95% CI] in Experiment 2. Parameter *s* = probability of successful target storage, $r_1$ = probability of successful target retrieval during recognition, *g* = probability of guessing ’old’ during recognition, $r_2$ = probability of successful target retrieval during recall. For the aggregated data, the model was fitted using ML estimation in the R package MPTinR^31^ (95% confidence intervals in brackets), and parameter $r_1$ was set equal between the waking rest and the social media condition to allow for a model fit evaluation. For the individual data, the model was fitted using Bayesian hierarchical estimation in the R package TreeBUGS^33^ (95% Bayesian credibility intervals in brackets).

Parameter	Waking rest	Social media	Vocabulary
Aggregated data			
Storage (*s*)	0.92 [0.90, 0.93]	0.92 [0.90, 0.94]	0.89 [0.87, 0.91]
Recognition retrieval ($r_1$)	1.00 [1.00, 1.00]	1.00 [1.00, 1.00]	0.99 [0.99, 1.00]
Guessing “old” (*g*)	0.41 [0.31, 0.51]	0.27 [0.18, 0.36]	0.27 [0.20, 0.35]
Cued recall retrieval ($r_2$)	0.63 [0.60, 0.67]	0.66 [0.63, 0.69]	0.63 [0.60, 0.67]
Individual data			
Storage (*s*)	0.92 [0.90, 0.94]	0.93 [0.91, 0.96]	0.89 [0.87, 0.92]
Recognition retrieval ($r_1$)	1.00 [0.99, 1.00]	1.00 [0.99, 1.00]	0.99 [0.98, 1.00]
Guessing “old” (*g*)	0.30 [0.12, 0.49]	0.29 [0.13, 0.47]	0.27 [0.14, 0.40]
Cued recall retrieval ($r_2$)	0.64 [0.59, 0.68]	0.66 [0.63, 0.70]	0.64 [0.59, 0.69]

## Discussion

In the present research, we set out to find direct evidence for a role of consolidation processes in the waking rest effect by replicating and extending the social media study reported by Martini et al.^7^.

Across both experiments, we failed to find any memory differences between the waking rest and social media conditions. Thereby, our results add to a growing body of evidence suggesting that certain distractor tasks might be equally beneficial for consolidation as waking rest^11^. Indeed, it has been argued that waking rest might not always minimize cognitive processing, but instead trigger highly active processes such as mentalizing, mind-wandering, and autobiographical thinking^42^. In contrast, past research has identified relaxation as an important motivational aspect of social media engagement^43^. Thus, the waking rest versus social media effect might be susceptible to how much participants engage in effortful cognitive processing during waking rest and social media use.

That said, the striking discrepancy between the original finding of a medium to large waking rest versus social media effect and our own null findings across two experiments might also be explained through procedural differences. Word pairs in our study were presented for 12 instead of just 5 seconds during the learning phase, and we applied a 35% learning criterion that was not used in the original study. This was necessary to ensure sufficiently high category frequencies in the storage-retrieval MPT model. Thereby, mean numbers of correct responses in the immediate cued recall were substantially increased in our experiments (*M* = 10.96−12.00) compared to what was found by Martini et al. (*M* = 7.64 in the waking rest condition, *M* = 7.21 in the social media condition). However, although we cannot rule out that a retroactive interference effect of social media use relative to a waking rest condition would replicate for weaker forms of initial learning, we think it is important to note that such an effect disappears given stronger forms of initial learning as employed here.

This would be in line with the suggestion that encoding strength moderates the waking rest effect^11^, such that the effect might be reduced or even eliminated for relatively long presentation times^44^. Conceivably, higher encoding strength might decrease the necessity for consolidation, especially for rather short retention intervals of a few minutes. Such an explanation might in principle apply to our results. Importantly, however, the reliable storage impairment in the vocabulary condition (see below) suggests that even in case of relatively high encoding strength, consolidation can still be disrupted by some forms of post-encoding mental activity. Future studies may further scrutinize this issue by systematically manipulating the learning criterion and observing its influence on the waking rest effect and MPT parameter estimation precision. For the time being, replication failures such as ours call into question the use of waking rest interventions in relevant applied settings.

We did find reliable differences in cued recall retention and recognition performance scores between the waking rest and vocabulary conditions across both experiments. Disregarding our model-based results, such a finding could be easily explained by more traditional accounts of similarity-based retroactive interference that attribute retroactive interference effects to some form of response competition or indiscriminability at the retrieval stage^15,45^. Indeed, such accounts could be reconciled with our result pattern for cued recall performances under the assumption of no or relatively low similarities between the Icelandic-German vocabulary and social media content. However, our storage-retrieval MPT analyses revealed that the differences between the vocabulary condition and the waking rest and social media conditions were driven solely by storage processes. In other words, our results are at odds with traditional explanations of retroactive interference. Instead, we found first direct behavioral evidence for a role of consolidation in the waking rest effect.

Our result pattern with respect to MPT storage probabilities $s$ suggests that some diversion threshold needs to be reached before consolidation processes are inhibited. Apparently, only the very high degree of intentional encoding demands induced in the vocabulary condition was sufficient to interfere with consolidation. This observation contradicts the original key assumption by Dewar et al.^3^ according to which any interpolated material or task that induces cognitive processing and encoding demands beyond mere waking rest will interfere with consolidation. However, more research is needed to determine whether or not such storage effects can also be observed for low similarity distractor tasks. An ideal candidate to use in such an investigation might be the d2 test of attention^46^, that is, a highly controlled attention and concentration performance test for which negative effects compared to waking rest have recently been demonstrated for some participants^41,47^. Such a non-verbal task can be assumed to share virtually no similarities with the original learning task while inducing considerable cognitive processing demands.

In principle, our finding of a difference in MPT storage probabilities $s$ between the waking rest and social media conditions on the one hand and the vocabulary condition on the other hand could also be attributed to systematic differences in the use of mnemonic strategies such as active rehearsal of the Icelandic-German vocabulary during the retention interval. However, such a confounding influence should have been reflected not only in storage probabilities $s$ but also in cued recall retrieval probabilities $r_2$. As we did not observe clear evidence for such a retrieval effect in both experiments, the storage differences between conditions can most likely be explained by differences in consolidation. This conclusion is further supported by our sensitivity analyses (see Supplementary Tables S4, S6, and S8).

It should be acknowledged that our current procedural approach cannot discriminate whether waking rest actively facilitates consolidation or passively protects newly created memory traces from the damaging influence of diversion retroactive interference. Future research may apply the storage-retrieval MPT approach to an alternative paradigm where equivalent levels of retroactive interference are ensured in all conditions but the timing of the distractor task during the retention interval is manipulated^5,6^. Under such conditions, all newly created memory traces have to endure the same absolute level of retroactive interference. Thus, if our model-based results could be replicated in this paradigm, an active facilitation effect of waking rest might appear more plausible than a passive protection effect. However, such a reasoning would rest on the critical assumption that damaging effects of retroactive interference do not follow a temporal gradient, a notion that has been disputed by prior research^45,48^.

Our mixed findings in Experiment 1 and clear null-findings in Experiment 2 for cued recall retrieval probabilities $r_2$ lead to a rather complex conclusion with respect to similarity retroactive interference. Apparently, the similarities between Icelandic-German and unrelated Norwegian-German word pairs were insufficient to result in significant retrieval competition^49^ or indistinctiveness^45,48^ during the delayed memory tests. One reason for this might be that our testing procedure did not involve free recall tests, that is, participants were always presented with some retrieval cue: either the cue word of the respective word pair (cued recall) or even the target word itself (recognition). It seems likely that retrieval differences would have been more pronounced had we used a testing procedure involving free recall. Future research might use alternative storage-retrieval MPT models that involve such free recall tests^27,50^.

To our surprise, we found mixed evidence for an effect of our manipulation on guessing probabilities $g$ during recognition. Descriptively, the probability of guessing ‘old’ was highest in the waking rest and lowest in the vocabulary condition across both experiments. Although this pattern was only reliable in Experiment 1, our results at least tentatively suggest that positive effects of post-encoding waking rest on recognition performances might be partially explained by a more balanced guessing style (i.e., $g$ closer to 0.5 in case of equal numbers of targets and distractors) in the waking rest condition compared to an overly conservative guessing style (i.e., $g$ closer to 0.0) in distractor conditions.

Overall, the MPT result patterns largely aligned between both experiments, suggesting that parameter estimates resulting from this model are rather robust against changes to the test order. However, mean cued recall retention scores above 1 in all three conditions in Experiment 1 suggest that participants benefited from being presented with all target words during the preceding recognition test. In contrast, in Experiment 2, the reversal of the memory testing procedure led to a reduction in cued recall retention scores. Thus, the overall performance level in Experiment 2 might be more trustworthy.

Using storage-retrieval MPT modeling allowed us to directly measure consolidation contributions to memory performance on a behavioral level. We hope that future research on the positive effects of post-encoding waking rest will adopt such a model-based approach. We are optimistic that the field will thereby reach an even more comprehensive and nuanced view of the respective roles of diversion and similarity retroactive interference in the waking rest effect.

## Supplementary Information

Supplementary Information.

## Data Availability

The datasets generated during the current studies and R scripts necessary to reproduce all reported results are available on the OSF at 10.17605/OSF.IO/K2GS8.
