# Supplementary material for: Waking rest during retention facilitates memory consolidation, but so does social media use
Source: Sci Rep. 2025 Feb 18;15:5884. doi: 10.1038/s41598-025-88363-z (PMC11836141; doi:10.1038/s41598-025-88363-z)
Supplement: Supplementary file 1 — Supplementary Information. [file 41598_2025_88363_MOESM1_ESM.pdf]

Supplementary Information

accompanying the manuscript

Waking rest during retention facilitates memory consolidation, but so does social media use

Main cued recall and recognition analysis of Session 2 in Experiment 1

Our expectations with respect to cued recall and recognition measures in Session 2 of Experiment 1 were the same as for Session 1. We observed no significant overall effect of our manipulation on the number of correct responses in the second delayed cued recall, $F(2, 138) = 1.96$, $p = 0.145$, $\eta^2 = 0.03$, or on the cued recall retention across the 24-hr interval between sessions, $F(2, 138) = 1.79$, $p = 0.171$, $\eta^2 = 0.03$. In line with this, planned contrasts revealed no significant difference between the waking rest and the social media condition, $t(138) = 0.78$, $p = 0.220$, Cohen's $d = 0.16$. Importantly, however, cued recall retention in the vocabulary condition was significantly lower than in the waking rest condition, $t(138) = 1.88$, $p = 0.031$, Cohen's $d = 0.39$. There was no significant difference (two-tailed) between the social media and the vocabulary condition, $t(138) = 1.13$, $p = 0.259$, Cohen's $d = 0.24$.

Recognition performances in Session 2 were overall not significantly affected by our manipulation, $F(2, 138) = 1.88$, $p = 0.157$, $\eta^2 = 0.03$. Accordingly, planned contrasts revealed no significant differences between the waking rest and the social media condition, $t(138) = -0.46$, $p = 0.676$, Cohen's $d = -0.09$, or between the waking rest and the vocabulary condition, $t(138) = 1.43$, $p = 0.078$, Cohen's $d = 0.30$. There was also no significant difference (two-tailed) between the social media and the vocabulary condition, $t(138) = 1.87$, $p = 0.064$, Cohen's $d = 0.39$.

Measure	Waking rest	Social media	Vocabulary
Second delayed recall: Correct responses (absolute)	13.29 (3.11)	13.35 (4.08)	12.02 (3.45)
Second delayed recall: Retention	1.15 (0.21)	1.12 (0.24)	1.07 (0.19)
Second delayed recognition: Hit rate	0.94 (0.07)	0.93 (0.08)	0.90 (0.09)
Second delayed recognition: False-alarm rate	0.04 (0.05)	0.02 (0.04)	0.03 (0.05)
Second delayed recognition: Performance	0.89 (0.09)	0.90 (0.08)	0.87 (0.10)

Supplementary Table S1. Mean (*SD*) cued recall and recognition performances in the main analysis of Session 2 in Experiment 1. Recall retention = correct responses in the delayed cued recall / correct responses in the immediate cued recall. Recognition performance = hit rate − false-alarm rate. Recall proportions can be derived by dividing the absolute values by 20 (i.e., the number of stimuli in the cued recall tests).

Main Bayesian hierarchical MPT analysis of Session 1 in Experiment 1

The model fit the individual data from Session 1 well (p_{T1} = 0.502, p_{T2} = 0.549 in the waking rest condition, p_{T1} = 0.509, p_{T2} = 0.491 in the social media condition, p_{T1} = 0.502, p_{T2} = 0.521 in the vocabulary condition).

Storage probabilities s in Session 1 were not reliably higher in the waking rest than in the social media condition, Bayesian p = 0.530. In contrast, storage probabilities were reliably higher in the waking rest than in the vocabulary condition, Bayesian p = 0.029. There was also a reliable difference in storage probabilities between the social media and the vocabulary condition, 95% BCI = [0.00, 0.10].

Cued recall retrieval probabilities r_2 in Session 1 were not reliably higher in the waking rest than in the social media condition, Bayesian p = 0.631.

Guessing probabilities g in Session 1 differed reliably between the waking rest and the vocabulary condition, 95% BCI = [0.14, 0.62], but not between the waking rest and the social media condition, 95% BCI = [-0.05, 0.49], or the social media and the vocabulary condition, 95% BCI = [-0.04, 0.34].

Main MPT analysis of Session 2 in Experiment 1

In Session 2 of Experiment 1, storage probabilities s did not differ reliably between any conditions, $z = 0.51$, $p = 0.695$, Bayesian $p = 0.450$ (waking rest versus social media), $z = 1.15$, $p = 0.125$, Bayesian $p = 0.190$ (waking rest versus vocabulary), $\Delta G^2(1) = 2.68$, $p = 0.101$, 95% BCI = [-0.03, 0.06] (social media versus vocabulary). In contrast, the pattern for cued recall retrieval probabilities r_2 was the same as in Session 1, $z = 0.09$, $p = 0.465$, Bayesian $p = 0.624$ (waking rest versus social media), $z = 2.30$, $p = 0.011$, Bayesian $p = 0.081$ (waking rest versus vocabulary), $z = 2.26$, $p = 0.012$, Bayesian $p = 0.072$ (social media versus vocabulary). Again, recognition retrieval parameters r_1 were estimated to be very close to 1. The pattern for guessing probabilities g was also very similar to that in Session 1, $\Delta G^2(1) = 5.67$, $p = 0.017$, 95% BCI = [-0.05, 0.40] (waking rest versus social media), $\Delta G^2(1) = 11.34$, $p = 0.001$, 95% BCI = [0.07, 0.42] (waking rest versus vocabulary), $\Delta G^2(1) = 0.74$, $p = 0.391$, 95% BCI = [-0.14, 0.29] (social media versus vocabulary).

Parameter	Waking rest	Social media	Vocabulary
	Aggregated data		
Storage (s)	0.91 [0.89, 0.93]	0.91 [0.89, 0.93]	0.89 [0.86, 0.91]
Recognition retrieval (r_1)	0.99 [0.98, 1.00]	0.99 [0.98, 1.00]	0.98 [0.96, 0.99]
Guessing “old” (g)	0.41 [0.32, 0.50]	0.25 [0.17, 0.34]	0.20 [0.13, 0.28]
Recall retrieval (r_2)	0.73 [0.70, 0.77]	0.73 [0.70, 0.76]	0.68 [0.64, 0.71]
	Individual data		
Storage (s)	0.93 [0.89, 0.96]	0.92 [0.90, 0.95]	0.91 [0.87, 0.94]
Recognition retrieval (r_1)	0.99 [0.97, 1.00]	0.99 [0.98, 1.00]	0.98 [0.96, 0.99]
Guessing “old” (g)	0.39 [0.26, 0.52]	0.21 [0.05, 0.41]	0.14 [0.04, 0.28]
Recall retrieval (r_2)	0.74 [0.70, 0.79]	0.76 [0.69, 0.82]	0.69 [0.63, 0.75]

Supplementary Table S2. Storage-retrieval multinomial processing tree (MPT) parameter estimates (95% CI) in the main analysis of Session 2 in Experiment 1. s = probability of successful target storage, r_1 = probability of successful target retrieval during recognition, g = probability of guessing 'old' during recognition, r_2 = probability of successful target retrieval during recall. For the aggregated data, the model was fitted using ML estimation (95% confidence intervals in brackets), and parameter r_1 was set equal between the waking rest and the social media condition to allow for a model fit evaluation. For the individual data, the model was fitted using Bayesian hierarchical estimation (95% Bayesian credibility intervals in brackets).

Sensitivity analysis of Sessions 1 and 2 in Experiment 1

We conducted a sensitivity analysis to check the robustness of our main conclusions from Experiment 1. For Session 1, the respective analyses were conducted without the data from those participants who reported in the post-experimental questionnaire to have consciously rehearsed any of the Icelandic-German vocabulary during the 8-min retention interval (n = 55). For Session 2, they were conducted without the data from those participants who reported (a) 4 hours of sleep or less during the night between sessions (n = 2), (b) alcohol consumption between sessions (n = 31), (c) conscious rehearsal of the Icelandic-German vocabulary during the 24-hr interval between sessions (n = 55), (d) correct assumptions about our hypotheses (n = 1), or (e) a lack of understanding of the study instructions (n = 0). Overall, data from N_1 = 99 participants was included in the sensitivity analysis for Session 1 (n = 30 in the waking rest condition, n = 38 in the social media condition, n = 31 in the vocabulary condition), and data from N_2 = 65 participants in the sensitivity analysis for Session 2 (n = 29 in the waking rest condition, n = 23 in the social media condition, n = 13 in the vocabulary condition).

Descriptive statistics for cued recall and recognition performances are provided in Table S3 for Session 1 and in Table S5 for Session 2, MPT parameter estimates from both estimation approaches are provided in Table S4 for Session 1 and in Table S6 for Session 2. With respect to our dependent variables of main interest (i.e., cued recall retention, recognition performance, storage probability s, cued recall retrieval probability r_2), the overall data patterns largely aligned with those obtained from the main analyses. This tentatively indicates that our main conclusions are robust against conscious rehearsal, sleep deprivation, alcohol consumption, and participants' awareness of our hypotheses. However, given the considerably smaller sample sizes in the sensitivity compared to the main analyses, we deemed further significance testing uncalled-for. Thus, any data patterns observed in the sensitivity analysis should be treated with caution.

Measure	Waking rest	Social media	Vocabulary
Immediate recall: Repetitions	1.40 (0.56)	1.39 (0.50)	1.32 (0.54)
Immediate recall: Correct responses (absolute)	11.83 (2.67)	11.42 (3.39)	10.84 (2.45)
First delayed recall: Correct responses (absolute)	13.70 (3.10)	12.82 (3.89)	11.71 (3.49)
First delayed recall: Retention	1.17 (0.13)	1.13 (0.18)	1.07 (0.17)
First delayed recognition: Hit rate	0.95 (0.07)	0.94 (0.06)	0.86 (0.12)
First delayed recognition: False-alarm rate	0.05 (0.07)	0.03 (0.05)	0.03 (0.05)
First delayed recognition: Recognition performance	0.90 (0.11)	0.91 (0.08)	0.83 (0.13)

Supplementary Table S3. Mean (*SD*) cued recall and recognition performances in the sensitivity analysis of Session 1 in Experiment 1. Recall retention = correct responses in the delayed cued recall / correct responses in the immediate cued recall. Recognition performance = hit rate − false-alarm rate. Recall proportions can be derived by dividing the absolute values by 20 (i.e., the number of stimuli in the cued recall tests).

Parameter	Waking rest	Social media	Vocabulary
	Aggregated data		
Storage (s)	0.91 [0.89, 0.94]	0.93 [0.90, 0.95]	0.88 [0.85, 0.91]
Recognition retrieval (r_1)	0.98 [0.97, 0.99]	0.98 [0.97, 0.99]	0.94 [0.92, 0.97]
Guessing "old" (g)	0.48 [0.35, 0.60]	0.37 [0.26, 0.49]	0.17 [0.10, 0.24]
Recall retrieval (r_2)	0.75 [0.71, 0.79]	0.69 [0.66, 0.73]	0.67 [0.62, 0.71]
	Individual data		
Storage (s)	0.95 [0.90, 0.98]	0.93 [0.91, 0.96]	0.91 [0.86, 0.96]
Recognition retrieval (r_1)	0.99 [0.96, 1.00]	0.99 [0.97, 1.00]	0.98 [0.94, 1.00]
Guessing "old" (g)	0.49 [0.25, 0.74]	0.31 [0.13, 0.50]	0.12 [0.03, 0.24]
Recall retrieval (r_2)	0.75 [0.70, 0.81]	0.71 [0.64, 0.78]	0.67 [0.59, 0.75]

Supplementary Table S4. Storage-retrieval multinomial processing tree (MPT) parameter estimates [95% CI] in the sensitivity analysis of Session 1 in Experiment 1. s = probability of successful target storage, r_1 = probability of successful target retrieval during recognition, g = probability of guessing 'old' during recognition, r_2 = probability of successful target retrieval during recall. For the aggregated data, the model was fitted using ML estimation (95% confidence intervals in brackets), and parameter r_1 was set equal between the waking rest and the social media condition to allow for a model fit evaluation. For the individual data, the model was fitted using Bayesian hierarchical estimation (95% Bayesian credibility intervals in brackets).

Measure	Waking rest	Social media	Vocabulary
Second delayed recall: Correct responses (absolute)	13.48 (2.89)	11.78 (3.59)	12.38 (3.50)
Second delayed recall: Retention	1.17 (0.22)	1.11 (0.28)	1.09 (0.17)
Second delayed recognition: Hit rate	0.94 (0.06)	0.91 (0.09)	0.87 (0.10)
Second delayed recognition: False-alarm rate	0.04 (0.05)	0.03 (0.04)	0.02 (0.03)
Second delayed recognition: Performance	0.90 (0.08)	0.88 (0.08)	0.85 (0.10)

Supplementary Table S5. Mean (*SD*) cued recall and recognition performances in the sensitivity analysis of Session 2 in Experiment 1. Recall retention = correct responses in the delayed cued recall / correct responses in the immediate cued recall. Recognition performance = hit rate − false-alarm rate. Recall proportions can be derived by dividing the absolute values by 20 (i.e., the number of stimuli in the cued recall tests).

Parameter	Waking rest	Social media	Vocabulary
	Aggregated data		
Storage (s)	0.92 [0.89, 0.94]	0.89 [0.86, 0.92]	0.87 [0.82, 0.91]
Recognition retrieval (r_1)	0.99 [0.98, 1.00]	0.99 [0.98, 1.00]	0.99 [0.97, 1.01]
Guessing “old” (g)	0.37 [0.25, 0.49]	0.24 [0.13, 0.35]	0.11 [0.01, 0.20]
Recall retrieval (r_2)	0.74 [0.70, 0.78]	0.66 [0.61, 0.71]	0.72 [0.65, 0.78]
	Individual data		
Storage (s)	0.93 [0.89, 0.97]	0.90 [0.85, 0.93]	0.88 [0.79, 0.95]
Recognition retrieval (r_1)	0.98 [0.96, 1.00]	0.99 [0.97, 1.00]	0.98 [0.95, 1.00]
Guessing “old” (g)	0.36 [0.20, 0.53]	0.22 [0.03, 0.54]	0.12 [0.01, 0.37]
Recall retrieval (r_2)	0.74 [0.68, 0.80]	0.67 [0.58, 0.75]	0.73 [0.59, 0.85]

Supplementary Table S6. Storage-retrieval multinomial processing tree (MPT) parameter estimates [95% CI] in the sensitivity analysis of Session 2 in Experiment 1. s = probability of successful target storage, r_1 = probability of successful target retrieval during recognition, g = probability of guessing 'old' during recognition, r_2 = probability of successful target retrieval during recall. For the aggregated data, the model was fitted using ML estimation (95% confidence intervals in brackets), and parameter r_1 was set equal between the waking rest and the social media condition to allow for a model fit evaluation. For the individual data, the model was fitted using Bayesian hierarchical estimation (95% Bayesian credibility intervals in brackets).

Main Bayesian hierarchical MPT analysis of Experiment 2

The model fit the individual data well (p_{T1} = 0.501, p_{T2} = 0.501 in the waking rest condition, p_{T1} = 0.461, p_{T2} = 0.533 in the social media condition, p_{T1} = 0.496, p_{T2} = 0.464 in the vocabulary condition).

Storage probabilities *s* were not reliably higher in the waking rest than in the social media condition, Bayesian p = 0.751. In contrast, storage probabilities were reliably higher in the waking rest than in the vocabulary condition, Bayesian p = 0.045. There was also a reliable difference in storage probabilities between the social media and the vocabulary condition, 95% BCI = [0.00, 0.08].

Cued recall retrieval probabilities r_2 in Session 1 were not reliably higher in the waking rest than in the social media condition, Bayesian p = 0.809. The same held true for the comparisons between the waking rest and the vocabulary condition, Bayesian p = 0.536, as well as between the social media and the vocabulary condition, Bayesian p = 0.235.

Guessing probabilities *g* did not differ reliably between the waking rest and the social media condition, 95% BCI = [-0.23, 0.26], the waking rest and the vocabulary condition, 95% BCI = [-0.19, 0.26], or the social media and the vocabulary condition, 95% BCI = [-0.18, 0.23].

Sensitivity analysis of Experiment 2

As in Experiment 1, we conducted a sensitivity analysis to check the robustness of our main conclusions. The respective analyses were conducted without the data from those participants who reported in the post-experimental questionnaire (a) to have consciously rehearsed any of the Icelandic-German vocabulary during the 8-min retention interval (n = 73), (b) correct assumptions about our hypotheses (n = 5), or (c) a lack of understanding of the study instructions (n = 0). Overall, data from N = 83 participants was included in the sensitivity analysis (n = 21 in the waking rest condition, n = 30 in the social media condition, n = 32 in the vocabulary condition).

Descriptive statistics for cued recall and recognition performances are provided in Table S7, MPT parameter estimates from both estimation approaches in Table S8. With respect to our dependent variables of main interest (i.e., cued recall retention, recognition performance, storage probability s, cued recall retrieval probability r_2), the overall data patterns largely aligned with those obtained from the main analyses. This indicates that our main conclusions are robust against conscious rehearsal and participants' awareness of our hypotheses. Again, sample sizes were considerably smaller than in the main analysis, so any data patterns observed in the sensitivity analysis should be treated with caution.

Measure	Waking rest	Social media	Vocabulary
Immediate recall: Repetitions	1.33 (0.58)	1.37 (0.61)	1.34 (0.65)
Immediate recall: Correct responses (absolute)	10.43 (2.91)	11.40 (2.92)	10.91 (2.79)
Delayed recall: Correct responses (absolute)	10.57 (2.89)	11.70 (2.96)	10.41 (3.19)
Delayed recall: Retention	1.02 (0.08)	1.03 (0.11)	0.95 (0.12)
Delayed recognition: Hit rate	0.95 (0.05)	0.93 (0.08)	0.90 (0.08)
Delayed recognition: False-alarm rate	0.05 (0.07)	0.01 (0.03)	0.03 (0.05)
Delayed recognition: Recognition performance	0.90 (0.09)	0.92 (0.09)	0.87 (0.08)

Supplementary Table S7. Mean (*SD*) cued recall and recognition performances in the sensitivity analysis of Experiment 2. Recall retention = correct responses in the delayed cued recall / correct responses in the immediate cued recall. Recognition performance = hit rate − false-alarm rate. Recall proportions can be derived by dividing the absolute values by 20 (i.e., the number of stimuli in the cued recall tests).

Parameter	Waking rest	Social media	Vocabulary
	Aggregated data		
Storage (s)	0.90 [0.87, 0.93]	0.92 [0.89, 0.94]	0.88 [0.85, 0.90]
Recognition retrieval (r_1)	1.00 [1.00, 1.00]	1.00 [1.00, 1.00]	0.99 [0.98, 1.00]
Guessing "old" (g)	0.46 [0.31, 0.61]	0.16 [0.06, 0.26]	0.26 [0.17, 0.35]
Recall retrieval (r_2)	0.59 [0.53, 0.64]	0.64 [0.60, 0.68]	0.59 [0.55, 0.64]
	Individual data		
Storage (s)	0.92 [0.87, 0.96]	0.93 [0.90, 0.96]	0.88 [0.85, 0.91]
Recognition retrieval (r_1)	0.99 [0.98, 1.00]	1.00 [0.99, 1.00]	0.99 [0.97, 1.00]
Guessing "old" (g)	0.40 [0.12, 0.69]	0.13 [0.02, 0.30]	0.23 [0.09, 0.39]
Recall retrieval (r_2)	0.58 [0.50, 0.66]	0.64 [0.58, 0.70]	0.60 [0.53, 0.66]

Supplementary Table S8. Storage-retrieval multinomial processing tree (MPT) parameter estimates (95% CI) in the sensitivity analysis of Experiment 2. s = probability of successful target storage, r_1 = probability of successful target retrieval during recognition, g = probability of guessing 'old' during recognition, r_2 = probability of successful target retrieval during cued recall. For the aggregated data, the model was fitted using ML estimation (95% confidence intervals in brackets), and parameter r_2 was set equal between the waking rest and the social media condition to allow for a model fit evaluation. A small positive constant of 0.10 was added to all category frequencies to avoid convergence issues due to categories with zero frequencies. For the individual data, the model was fitted using Bayesian hierarchical estimation (95% Bayesian credibility intervals in brackets).
